# The knowledge and attitudes of health professionals working in mother-friendly hospitals about complementary therapy and supportive care methods

**DOI:** 10.18332/ejm/146166

**Published:** 2022-04-13

**Authors:** Nursen Bolsoy, Esra Bozhan-Tayhan, Seçil Köken-Durgun, Elif Damar, Emine Kayıp

**Affiliations:** 1 Midwifery Department, Faculty of Health Sciences, Manisa Celal Bayar University, Manisa, Turkey; 2 Kula District Health Directorate, Ministry of Health, Manisa, Turkey; 3 Maternity Service, Manisa City Hospital, Ministry of Health, Manisa, Turkey

**Keywords:** mother friendly hospital, complementary, supportive, healthcare professionals, attitude

## Abstract

**INTRODUCTION:**

It is important that the healthcare professionals who are with the mother at the moment of birth and afterwards, know and apply effective complementary treatment and supportive care methods, and also know their effects and limitations. The aim of this study is to determine the knowledge levels and attitudes of health personnel about complementary therapy and supportive care methods to be used in the management of labor pain and postpartum period.

**METHODS:**

A cross-sectional study included 142 midwives/nurses and physicians working in the delivery room and maternity services of mother-friendly hospitals in Manisa, Turkey, between August 2018 and April 2019. The questionnaire consisting of 25 questions evaluated the knowledge and attitudes of the participants about complementary therapy and supportive care methods.

**RESULTS:**

It was found that 30.6% of the healthcare professionals participating in the study used complementary treatment and supportive care methods in their patients. The least heard method was chiropractic (6.6%), the best-known method was hydrotherapy (water birth) (5.8%) and the most used method was massage (14.0%). It was determined that the mean total attitude score of the healthcare workers on complementary treatment and supportive care methods was 18.57 ± 5.12 (range: 8–40). It was found that the education levels of healthcare professionals (z= -2.144, p<0.05) and the institutions affect (χ^2^=23.417; p<0.05) the attitude scores.

**CONCLUSIONS:**

One out of every two healthcare professionals did not have information about complementary treatment and supportive care methods. Healthcare professionals mostly have positive opinions about these methods.

## INTRODUCTION

Pregnancy and childbirth are normal, healthy and natural functions of the body, which are accepted as normal processes in most societies; childbirth is one of the best life experiences for parents^1^. However, negative experiences and stories about childbirth have caused birth to be remembered with pain and fear in society. This situation has led pregnant women to give birth by cesarean section^2^. In addition, the proliferation of unnecessary interventions in natural birth has mobilized the Coalition for Improving Maternity Services (CIMS), which works for the care and welfare of mothers, babies and families^3^.

The concept of mother-friendly birth was developed by CIMS in 1996, and it means a natural and healthier birth practice and care for mother and baby^3^. The purpose of care in the mother-friendly birth model is to support the development of maternal and child health with evidence-based practices^4^. For this purpose, principles that increase, protect and support mother-friendly birth services and mother-friendly practices in ten steps have been established. Since the relevant criteria are included in the publications on mother-friendly hospitals, this article will not mention mother-friendly practices in ten steps and the principle^3^. In addition, The Mother Baby-Family model is mentioned in the Safe and Respectful Mother Baby-Family Birth Care guide published by The International Childbirth Initiative (ICI) in cooperation with important non-governmental organizations, in line with mother-friendly practices. This model includes the health services provided to the mother and baby in the pre-pregnancy, pregnancy, birth and postnatal period. According to the model, there are 12 steps to be followed in the care to be given to the mother and baby^5^:

1. Provide respect, dignity and informed choice
2. Provide free or affordable care with cost transparency
3. Routinely provide mother and baby maternity care
4. Offer continuous support
5. Provide pain relief measures
6. Provide evidence-based practice
7. Avoid harmful practices
8. Enhance wellness and prevent illness
9. Provide emergency care and transport
10. Have a supportive human resource policy
11. Provide a care continuum
12. Promote breastfeeding and skin-to-skin contact^5^.

With mother-friendly practices, the common point reached today is to ensure that the birth is managed in the least possible attempt and in the healthiest way possible. Existing evidence suggests that midwives may consider complementary therapies compatible with the woman-centered midwifery philosophy^6^. In this process, depending on the criteria of the mother-friendly hospital, the mother’s wishes are listened to, and she is directed to decide on the method of delivery as she wishes. More attention was taken for care to the privacy of the mother, and routine practices that were not based on evidence were abolished. It provided the opportunity to walk and move comfortably in the room, which was proven to relieve the birth pain^2^. It also advocated the need for complementary therapy and supportive care for labor pain.

Supportive care has become increasingly important and supportive care practices have increased in all areas of health^7^. Also, various methods are used in the control of birth pain, such as massage, hand foot massage, phytotherapy, acupressure, aromatherapy, acupuncture, hot-cold application, moving, yoga, hypnotherapy, and changing position^8^. It is important for the healthcare professionals, who are with the mother at birth, to know these methods, their effects, limitations and to apply them effectively in controlling labor pain. When the literature was examined, it was found that there were studies showing that healthcare personnel did not have enough information about complementary therapy and supportive care^9-11^. Therefore, undergraduate and in-service training of midwives who are supportive of the mother at birth has gained importance. Before the education, it has been considered that it is necessary to determine the status of the information gap.

In order for health professionals to increase the use of mother-friendly practices, it is important to know their knowledge levels and attitudes towards complementary therapy and supportive care methods. In this study, it was aimed to determine the knowledge levels and attitudes of health professionals about complementary therapy and supportive care methods to be used in relieving labor pain and in the management of the postpartum period.

## METHODS

### Design and setting

This cross-sectional study was applied in the city of Manisa, Turkey. Manisa is an industrial city in western Turkey and 49.6% of the population are women. In Turkey, the switch to mother-friendly practices began with Manisa, with its selection as the first pilot province. This research was conducted between August 2018 and April 2019 in five public hospitals, one in the center and four in different districts, which received the title of Mother-Friendly Hospital in Manisa. In this study, the descriptive data of the first phase of the project named ‘The Effect of Supportive Care Methods Training Provided to Health Personnel Working in Mother-Friendly Hospitals on the Knowledge Levels and Attitudes of Health Personnel’ supported by Manisa Celal Bayar University Scientific Research Projects is shared.

### Sample size

The population of the study consisted of midwives/nurses and doctors working in the delivery rooms and maternity services of the relevant hospitals (n=142). The population could not be expanded further, as the study was conducted in hospitals that were named mother-friendly hospitals. It was aimed to reach the population without using the sample selection method. During the data collection process, midwives/nurses and doctors who left their jobs or were on annual leave, or did not agree to participate in the study, were excluded, and the study was completed with 121 healthcare professionals (participation rate 85.2%).

### Study instrument

A questionnaire consisting of 25 questions was used to collect data. The first 17 items of the questionnaire were prepared by the researchers in line with the literature. The Attitude Scale towards Complementary and Alternative Medicine, developed by Araz and Harlak^12^ in 2006, constituted the last 8-item part of the questionnaire. The scale was organized with responses on a 5-point Likert type (5=strongly agree, 4=agree, 3=neither agree nor disagree, 2=disagree, 1=strongly disagree). Some of the items (5, 6, 8) were randomly reversed. The range of the scores was possibly between 8 and 40. Higher scores meant positive attitudes. The Cronbach alpha value of the scale is 0.82. For this study it was calculated as 0.82.

### Data collection

This study was conducted according to the Declaration of Helsinki. Participants were informed about the research objectives and procedure, and their verbal and written consent was obtained. Institutions were visited by the researchers, and healthcare professionals were given questionnaires evaluating their knowledge and attitudes about complementary therapy and supportive care methods.

### Statistical analysis

The data obtained were evaluated using SPSS. In evaluating the data, percentage, mean, median, Kruskal Wallis test and Mann Whitney U test statistical analysis were used. A p<0.05 was considered statistically significant.

## RESULTS

The average age of the healthcare professionals participating in the study was 39.9 ± 6.4 years (range: 23–60). It was determined that 88.4% of the participants were women, 90.1% were married, and 51.2% were university graduates. Considering their professional characteristics, 14.0% were physicians, 74.4% were midwives, and 11.6% were nurses. The average working time of the healthcare professionals included in the study was 17.84 ± 7.78 years (range: 1–40), and the average working time in the maternity service or delivery room was 9.99 ± 7.70 (range: 1–30) (Table 1).

**Table 1:** Some descriptive and professional characteristics of healthcare professionals, Manisa, Turkey (N=121)

*Characteristics*	*Categories*	*n (%)*
Age (years) Mean ± SD: 39.95 ± 6.43 Range: 23-60	≤30	10 (8.3)
	31-40	59 (48.8)
	41-50	44 (36.4)
	≥51	8 (6.6)
Gender	Woman	107 (88.4)
	Male	14 (11.6)
Marital status	Married	109 (90.1)
	Single	12 (9.9)
Education level	High school	18 (14.9)
	Associate degree	19 (15.7)
	University	62 (51.2)
	Master’s degree	22 (18.2)
Profession	Physician	17 (14.0)
	Midwife	90 (74.4)
	Nurse	14 (11.6)
Institution	Merkez Efendi State Hospital	43 (35.5)
	Akhisar Mustafa Kirazoğlu State Hospital	19 (15.7)
	Turgutlu State Hospital	26 (21.5)
	Alaşehir State Hospital	17 (14.0)
	Soma State Hospital	16 (13.2)
Working time in the profession (years) Mean ± SD: 17.84 ± 7.78 Range: 1-40	1-10	25 (20.7)
	11-20	49 (40.5)
	21-30	44 (36.4)
	31-40	3 (2.5)
Working time in the maternity service and delivery room (years) Mean ± SD: 9.99 ± 7.70 Range: 1-30	1-10	71 (58.7)
	11-20	40 (33.1)
	21-30	10 (8.3)

Of the healthcare professionals, 77.7% had previously participated in in-service training on labor pain and postpartum care methods; of these 89.3% attended less than four in-service training sessions. It was determined that 49.6% of the healthcare professionals participating in the in-service training received information on the positive and negative effects of complementary treatment and supportive care methods on labor (Table 2).

**Table 2:** Healthcare professionals’ participation in in-service training on labor pain and postpartum care in the last 3 years, Manisa, Turkey (N=121)

	*Categories*	*n (%)*
Participation in in-service training	Yes	94 (77.7)
	No	27 (22.3)
Number of attending in-service training* Mean ± SD: 2.36 ± 1.47 Range: 1-8	≤4	67 (89.3)
	≥5	8 (10.7)
Information received in in-service training** (n=94)	Physiology of labor pain	72 (59.5)
	Pain theories	54 (44.6)
	Assessment of labor pain	69 (57.0)
	Factors causing pain at birth	67 (55.4)
	The effect of pain on labor	69 (57.0)
	The importance of labor pain control	75 (62.0)
	Positive and negative effects of pharmacological methods on labor	59 (48.8)
	Positive and negative effects of complementary treatment and supportive care methods on labor	60 (49.6)
	The role of the midwife/nurse in the management of labor	75 (62.0)
	Mother-baby attachment	72 (59.5)
	Breastfeeding	83 (68.6)
	Other	20 (16.5)

* 73 people answered.

** More than one option has been marked.

Of the healthcare professionals, 90.9% thought that complementary therapy and supportive care methods provide pain control and facilitate labor; 30.6% of the participants stated that they used the relevant methods and 88.4% that they would like to receive training on complementary therapy and supportive care methods (Table 3).

**Table 3:** Healthcare professionals’ opinions about complementary therapy and supportive care methods and their willingness to receive training, Manisa, Turkey (N=121)

*Opinions*		*n (%)*
Ideas about pain control and facilitation of labor	Yes	110 (90.9)
	No	11 (9.1)
Use of related methods	Yes	37 (30.6)
	No	84 (69.4)
Willingness to receive education	Yes	107 (88.4)
	No	10 (8.3)
	No idea	4(3.3)

Findings related to the knowledge and opinions of the healthcare professionals about complementary treatment and supportive care methods are given in Table 4.

**Table 4:** Healthcare professionals’ knowledge and opinions on complementary therapy and supportive care methods, Manisa, Turkey (N=121)

*Complementary therapy and supportive care methods*	*Knowledge and opinion*					
	*Never heard*	*Heard*	*Limited information*	*Enough information*	*Know the method in every way*	*Use it for my patients*
	*n (%)*	*n (%)*	*n (%)*	*n (%)*	*n (%)*	*n (%)*
Acupuncture	15 (12.4)	65 **(53.7)**	32 **(26.4)**	9 (7.4)	0 **(0.0)**	0 **(0.0)**
Aromatherapy	56 (46.3)	37 (30.6)	17 (14.0)	9 (7.4)	1 (0.8)	1 (0.8)
Reflexology	65 (53.7)	33 (27.3)	14 (11.6)	8 (6.6)	1 (0.8)	0 **(0.0)**
Phytotherapy	59 (48.8)	38 (31.4)	19 (15.7)	5 (4.1)	0 **(0.0)**	0 **(0.0)**
Therapeutic touch	58 (47.9)	32 (26.4)	20 (16.5)	8 (6.6)	2 (1.7)	1 (0.8)
Music	18 (14.9)	57 (47.1)	23 (19.0)	20 (16.5)	3 (2.5)	0 **(0.0)**
Acupressure	93 (76.9)	12 (9.9)	9 **(7.4)**	4 **(3.3)**	3 (2.5)	0 **(0.0)**
Homeopathy	86 (71.1)	12 (9.9)	15 (12.4)	7 (5.8)	1 (0.8)	0 **(0.0)**
Hypnotherapy	56 (46.3)	27 (22.3)	24 (19.8)	13 (10.7)	1 (0.8)	0 **(0.0)**
Chiropractic	98 **(81.0)**	8 **(6.6)**	9 **(7.4)**	6 (5.0)	0 **(0.0)**	0 **(0.0)**
Yoga	14 (11.6)	56 (46.3)	29 (24.0)	18 (14.9)	2 (1.7)	2 (1.7)
Positioning	13 (10.7)	36 (29.8)	24 (19.8)	29 (24.0)	4 (3.3)	15 (12.4)
Massage	5 **(4.1)**	31 (25.6)	29 (24.0)	33 (27.3)	6 (5.0)	17 **(14.0)**
Hydrotherapy (water birth)	9 (7.4)	50 (41.3)	26 (21.5)	22 (18.2)	7 **(5.8)**	7 (5.8)
Hot-cold application	8 (6.6)	45 (37.2)	21 (17.4)	30 **(24.8)**	2 (1.7)	15 (12.4)

Accordingly, chiropractic was found to be the unheard-of method for 6.6%, and acupuncture was the most commonly heard method with a rate of 53.7%. It was determined that the best-known method was hydrotherapy (water birth) with a rate of 5.8%, and the most used method was massage with a rate of 14.0%. It was determined that the mean total attitude score of the healthcare professionals on complementary therapy and supportive care methods was 18.57 ± 5.12 (range: 8–40).

The relationship between the descriptive and professional characteristics of the healthcare professionals and their total attitude scores was examined. There was no statistically significant difference between the independent variables of age, gender, marital status, profession, working time in the profession, working time in the maternity service and delivery room, and attitude scores (p>0.05). A statistically significant difference was found between the independent variables of education (z= -2.144, p<0.05), the institution (χ^2^=23.417; p<0.05) and their attitude scores.

The relationship between the healthcare professionals’ knowledge and use of complementary therapy and supportive care methods and their total attitude scores were examined. A statistically significant difference was found between the independent variable of providing pain control and facilitating delivery of the related methods and attitude scores (z= -3.015; p<0.05). There was no statistically significant difference between the attitude scores and the amount of information about the relevant subject, the willingness to receive education, and the use of methods (p>0.05).

## DISCUSSION

In addition to the treatment programs accepted by health professionals, the interest and use of many applications in order to protect, treat and improve health is increasing. Particularly because women generally attach more importance to their own healthcare and treatment in case of illness, the rate of using complementary applications is high^13^. At the same time, the usefulness of complementary therapy and supportive care applications for women’s health has been proven by numerous studies^14-19^. As a result of these studies, it is stated that it can increase fertility^14^, reduce the perception of pain at labor^15,16^, prevent the formation of postpartum breast disorders^17^, improve mood disorders in women receiving infertility treatment^18^, and have mitigating effects on problems present during menopause^19^. The use of complementary treatment and supportive care practices, which are beneficial in all stages of women’s life, by health service providers, should be widespread and supported.

The naturally progressing labor and labor process appear to be a much feared and badly remembered experience, where the pregnant woman does not take part in her labor, and with the developing technology, excessive medical intervention takes place^20^. Healthcare professionals have a lot of responsibility to break this taboo. It is essential to have sufficient knowledge about labor pain and postnatal care methods and develop a positive attitude about complementary and supportive care methods. Lafçı and Kaşıkçı^11^ examined healthcare professionals’ knowledge and use of complementary and alternative therapies methods in Turkey. As a result of the study, it was stated that the rate of healthcare professionals who took any course or training on related methods was only 4.8%. In this study, the rate of getting information about labor pain and postpartum care methods was found to be 77.7%, while only half of them (49.6%) were informed about complementary therapy and supportive care methods. Since the hospitals where the study was conducted are mother-friendly hospitals, it can be said that the knowledge and attitudes of healthcare professionals have increased positively thanks to the in-service training given about the related methods. However, it was still observed that one in two healthcare personnel did not have information about complementary therapy and supportive care methods.

Complementary therapy and supportive care methods are widely used at childbirth all over the world^6^. Such as acupuncture, acupressure, aromatherapy, homeopathy, phytotherapy, ozone therapy, oxygen therapy, mesotherapy, massage, hypnotherapy, ayurveda, therapeutic touch, yoga, cryotherapy, meditation, osteopathy, reflexology, spa therapy, thermal therapy, hydrotherapy, music therapy, positioning, and hot-cold applications^21^. Although there are many methods, very few are known by healthcare professionals^9-11^. In the study of Sen^9^ comparing the complementary alternative medicine methods used by healthcare professionals and non-healthcare professionals, the first three complementary treatment methods heard by healthcare professionals were hot-cold applications, acupuncture, and cupping; it has been reported that less than 20% of healthcare workers have heard of mesotherapy, apitherapy, osteopathy, neural therapy, and homeopathy practices. Çevik et al.^10^ examined nurses’ knowledge and views on complementary and alternative medicine in a study they planned. As a result of the study, it was reported that the first three applications that nurses have never heard of were chiropractic, shiatsu and homeopathy, and bioresonance, respectively. The first three practices of which they had sufficient knowledge were stated as dietary support, yoga, and meditation. Lafçı and Kaşıkçı^11^ stated that acupuncture, herbal therapy, dietary support and massage are the methods that healthcare professionals know the most about. In this study, acupuncture was found to be the most commonly heard method, and the unheard method was found to be chiropractic. It was determined that the best-known method in all aspects was hydrotherapy. Considering the results of the study, it is thought that healthcare professionals have more information about the methods that are popular today. In addition, according to the results of the study examined, it was seen that the occupational groups heard and knew almost the same methods. It is seen that the methods officially accepted in Turkey in the Regulation on Traditional and Complementary Medicine Practices (dated 2014 numbered 29152) slightly affect the knowledge of that method^22^. The reason why complementary therapy and supportive care methods are very low among healthcare professionals is thought to be due to the fact that these methods are not adequately processed in undergraduate or postgraduate education.

In order to provide holistic care in healthcare institutions, it is not enough to know complementary therapy and supportive care methods, and it is very important to use them^23^. While some of them are included in basic nursing/midwifery practices, the rate of using these methods, which require some special training before implementation, remains to be seen^24^. In the study of Bauer et al.^25^, in which they examined the attitudes of healthcare professionals towards complementary and integrative medicine in 2020, it was seen that they had a positive attitude. Shorofi and Arbon^26^ examined nurses’ knowledge, practices and attitudes about complementary and alternative medicine in Australia. As a result of the study, it was reported that 49.7% of the nurses used the relevant methods on their patients, and the most widely used method was massage. In the study conducted by Bahall and Legall^27^ to examine the knowledge, attitudes and practices of healthcare professionals about alternative medicine in Trinidad and Tobago, 82.3% of the participants used the methods, the rate of use was affected by gender, race and profession. Lafçı and Kaşıkçı^11^ stated that a quarter of the healthcare professionals they included in the study used at least one of the complementary therapy and supportive care methods. They reported that herbal therapy and massage took the first place in use. According to the results of the study conducted by Wardle et al.^28^ to examine the referral behaviors of general practitioners to physicians practicing complementary and alternative medicine, only 2.7% of general practitioners refer patients once a month. Samuels et al.^29^ found that most midwives and nurses (87.3%) used these methods in the study in which nurses and midwives working in the maternity services in Israel evaluated the use of complementary and alternative medicine and their attitudes on this issue. It was reported that the most used methods in the study were massage, herbal medicine, meditation/yoga. Only 30.6% of the participants in this study stated that they used these methods. While almost all of them thought that the applications will facilitate birth and increase pain control, the rate of practicing was very low. The methods that were never used were found to be acupuncture, reflexology, phytotherapy, music, acupressure, homeopathy, hypnotherapy, and chiropractic. When the results of the study are evaluated, it is seen that the methods used are similar, while the rate of using the methods is higher in some countries. This can be explained by the deciding on the usefulness of the relevant methods and how widespread their use was before. In Turkey, with the increase of information exchange, it will be supported and their usage will be widespread. However, it is thought that education and certification programs approved by the Ministry of Health are required for this.

The majority of the participants mentioned the importance of in-service training in order to spread the use of complementary therapy and supportive care methods among healthcare professionals. In addition, it is highly recommended to provide information to prenatal pregnant women about these methods and to provide a suitable environment and materials for the application. In this way, it is thought that the problems encountered, such as rejection of the method by the pregnant woman or the inability to apply the method, due to a lack of material, will be reduced.

Attitudes towards these methods as well as the training given to healthcare professionals on complementary therapy, are also very effective in their practice. As a result of the study of Shorofi and Arbon^26^, 22.4% of the nurses had a very positive attitude, and 36.6% had a partially positive attitude towards complementary therapy and supportive care methods. In the study of Bahall and Legall^27^, 62.3% of doctors and 64.0% of nurses were shown to have a positive attitude that complementary therapy and supportive care methods would improve health. In the study of Samuels et al.^29^, most of the participants gave high scores to items indicating that traditional medicine could benefit from ideas and methods, and the average attitude score was found to be high, and their attitudes were found to be positive. In this study, it was found that the attitude of healthcare professionals was positive. It was determined that the independent variables such as the education of healthcare professionals, their institution, related methods that provide pain control and the opinion of facilitated birth, positively affected the attitude scores. It is thought that this situation may arise from having sufficient knowledge to apply complementary therapy and supportive care methods and having a suitable environment to practice. Although the number of doctors participating in the study is small (n=17), the fact that the average attitude scores are higher than the nurses and midwives shows that the education received affects the attitude. In the study of Bahall and Legal^27^, which supports the results of this study, doctors were more interested in obtaining information about complementary therapies compared to other healthcare professionals. It is thought that the difference in attitude points between the doctor and the midwife/nurse is also influenced by the autonomy of the applications. Despite the fact that the midwives-to-population ratio in Turkey is in a better position now than many OECD countries, midwife autonomy initiatives are low and not supported by existing legislation^30^. Turkey Regulation No. 29152 dated 2014 authorizes the use of methods on behalf of physicians^22^. This reduces the interest of midwives and nurses on the subject. For this reason, the application of related methods and their attitude towards these methods are lower. The professional independence of midwives/nurses and their competence in applying complementary therapies and supportive care methods need to be increased in order for the mother-friendly hospital practices to reach their full goals and to increase maternal and infant health.

## CONCLUSIONS

In this study, it was seen that the best-known method used by healthcare professionals working in mother-friendly hospitals was hydrotherapy (water birth), and the most used method was massage. It has been concluded that healthcare professionals mostly have positive opinions about complementary therapies and supportive care methods. In order to support this positive attitude, necessary planning should be made, considering the contribution of in-service training. It is considered necessary to include complementary therapies and supportive care methods in undergraduate and postgraduate in-service training to provide the highest standards of care in order to improve maternal and postnatal health. In addition, it is important to support midwives and nurses with regulations on implementation and to increase scientific studies that will create evidence.

## Data Availability

The data supporting this research are available from the authors on reasonable request.

## References

[1] Şahin M, Erbil N (2019). Doğum ve Medikalizasyon. Ordu Üniversitesi Hemşirelik Çalışmaları Dergisi.

[2] Serçekuş Ak P, Vardar O, Özkan S (2018). ANNE DOSTU HASTANELERİN YAYGINLAŞMASI TÜRKİYE İÇİN NEDEN ÖNEMLİDİR?. Necmettin Erbakan Üniversitesi Sağlık Bilimleri Fakültesi Dergisi.

[3] Mother-Friendly Childbirth Initiative (2016). http://www.motherfriendly.org/MFCI.

[4] Jukelevics N (2007). The Coalition for Improving Maternity Services: Evidence Basis for the Ten Steps of Mother-Friendly Care. The Foundation for Promoting Change in Maternity Care. Midwifery Today Int Midwife.

[5] (2020). The 12 Steps (summary version) to Safe and Respectful MotherBaby-Family Maternity Care.

[6] (2018). WHO recommendations: intrapartum care for a positive childbirth experience.

[7] Rathfisch G (2012). Doğal doğum felsefesi: milyonlarca yıldır gerçekleşen serüven.

[8] Berkiten Ergin A, Kömürcü N (2013). Doğum ve doğum tarihi. Doğum Ağrısı ve Yönetimi.

[9] Şen R (2017). Sağlık çalışanı olan ve olmayan ebeveynlerin çocuklarında uyguladıkları tamamlayıcı alternatif tıp yöntemleri.

[10] Çevik K, Bolsoy N, Beler M (2016). HEMŞİRELERİN TAMAMLAYICI VE ALTERNATİF TEDAVİYE İLİŞKİN BİLGİ VE GÖRÜŞLERİ. Uluslararası Hakemli Hemşirelik Araştırmaları Dergisi.

[11] Lafçı D, Kara Kasıkçı M (2014). YATAKLI SAĞLIK KURULUġUNDA GÖREV YAPAN SAĞLIK PERSONELĠNĠN TAMAMLAYICI VE ALTERNATĠF TEDAVĠ YÖNTEMLERĠNĠ BĠLME VE KULLANMA DURUMLARI. Gümüşhane Üniversitesi Sağlık Bilimleri Dergisi.

[12] Araz A, Harlak H (2006). Developing a scale for attitudes towards complementary and alternative medicine. Turkish journal of public health.

[13] Ilgaz A, Gözüm S (2016). Tamamlayıcı Sağlık Yaklaşımlarının Güvenilir Kullanımı için Sağlık Okuryazarlığının Önemi. Dokuz Eylül Üniversitesi Hemşirelik Fakültesi Elektronik Dergisi.

[14] Njamen D, Mvondo MA, Djiogue S, Ketcha Wanda GJM, Magne Nde CB, Vollmer G (2013). Phytotherapy and Women's Reproductive Health: The Cameroonian Perspective. Planta Med.

[15] Mallen-Perez L, Roé-Justiniano MT, Colomé Ochoa N, Ferre Colomat A, Palacio M, Terré-Rull C (2018). Use of hydrotherapy during labour: Assessment of pain, use of analgesia and neonatal safety. Enferm Clin (Engl Ed).

[16] Hamdamian S, Nazarpour S, Simbar M, Hajian S, Mojab F, Talebi A (2018). Effects of aromatherapy with Rosa damascena on nulliparous women's pain and anxiety of labor during first stage of labor. J Integr Med.

[17] Crepinsek MA, Taylor EA, Michener K, Stewart F (2020). Interventions for preventing mastitis after childbirth. Cochrane Database Syst Rev.

[18] Taguchi R, Sato K, Adomi S, Tanaka N, Tamura H, Tamura T (2019). Acupuncture and Laser Acupuncture as Treatments for Emotional Distress in Infertile Women in Japan. Med Acupunct.

[19] Khadivzadeh T, Najafi MN, Ghazanfarpour M, Irani M, Dizavandi FR, Shariati K (2018). Aromatherapy for Sexual Problems in Menopausal Women: A Systematic Review and Meta-analysis. J Menopausal Med.

[20] Aydın N, Yıldız H (2018). Effects of traumatic birth experience and transmission intergenerational. International Journal of Human Sciences.

[21] Biçer İ, Yalçin Balçik P (2019). GELENEKSEL VE TAMAMLAYICI TIP: TÜRKİYE VE SEÇİLEN ÜLKELERİNİN İNCELENMESİ. Hacettepe Sağlık İdaresi Dergisi.

[22] (2014). GELENEKSEL VE TAMAMLAYICI TIP UYGULAMALARI YÖNETMELİĞİ.

[23] Şahin N, Aydın D, Akay B (2019). HEMŞİRELİK ÖĞRENCİLERİNİN BÜTÜNCÜL TAMAMLAYICI VE ALTERNATİF TIBBA KARŞI TUTUMLARININ DEĞERLENDİRİLMESİ. Balikesir Sağlik Bilimleri Dergisi.

[24] Aktaş B (2017). Hemşirelik Öğrencilerinin Bütüncül Tamamlayıcı ve Alternatif Tıbba Karşı Tutumları. Hemşirelik Akademik Araştırma Dergisi.

[25] Bauer BA, Townsend KM, Cutshall SM (2020). Advanced Practice Providers' Knowledge, Attitudes, and Utilization of Complementary and Integrative Medicine at an Academic Medical Center. Altern Ther Health Med.

[26] Shorofi SA, Arbon P (2017). Complementary and alternative medicine (CAM) among Australian hospital-based nurses: knowledge, attitude, personal and professional use, reasons for use, CAM referrals, and socio-demographic predictors of CAM users. Complement Ther Clin Pract.

[27] Bahall M, Legall G (2017). Knowledge, attitudes, and practices among health care providers regarding complementary and alternative medicine in Trinidad and Tobago. BMC Complement Altern Med.

[28] Wardle JL, Sibbritt DW, Adams J (2013). Referral to Chinese medicine practitioners in Australian primary care: a survey of New South Wales rural and regional general practitioners. Chin Med.

[29] Samuels N, Zisk-Rony RY, Singer SR (2010). Use of and attitudes toward complementary and alternative medicine among nurse-midwives in Israel. Am J Obstet Gynecol.

[30] Health Care Resources: Midwives.

